# Non-Susceptibility of Early-Onset Sepsis Pathogens to the Combination of Ampicillin and Gentamicin Among Neonates in Thailand

**DOI:** 10.3390/antibiotics14050519

**Published:** 2025-05-17

**Authors:** Anucha Thatrimontrichai, Pattima Pakhathirathien, Manapat Praditaukrit, Gunlawadee Maneenil, Supaporn Dissaneevate

**Affiliations:** Division of Neonatology, Department of Pediatrics, Faculty of Medicine, Prince of Songkla University, Songkhla 90110, Thailand; ppattima@medicine.psu.ac.th (P.P.); manapat.p@psu.ac.th (M.P.); mgunlawa@medicine.psu.ac.th (G.M.); dsupapor@medicine.psu.ac.th (S.D.)

**Keywords:** *Acinetobacter baumannii*, ampicillin resistance, bacterial multidrug resistance, neonatal sepsis, newborn

## Abstract

**Background/Objectives**: Empirical antimicrobial therapy for neonatal early-onset sepsis (EOS) comprises ampicillin and gentamicin. However, multidrug-resistant organisms are increasing worldwide, thus inflicting a global burden. We identified the incidence and risk factors of neonates with pathogenic isolates that were not susceptible to treatment comprising a combination of ampicillin and gentamicin (non-susceptible group). **Methods**: This retrospective study included neonates diagnosed with EOS between 2004 and 2023. All patients with EOS and positive culture results within 72 h of birth were reviewed. Patients in the non-susceptible and susceptible groups were analyzed using a multivariable logistic regression model. **Results**: Sixty pathogenic isolates and 55 neonates with EOS were observed over the course of 20 years. The incidence and case fatality rates of EOS were 0.88 per 1000 live births and 41.8%, respectively. *Acinetobacter baumannii* was the most common EOS pathogenic isolate (19/60 pathogenic isolates; 12/19 resistant to carbapenems). Pathogenic isolates were susceptible to ampicillin or gentamicin (59%), ampicillin or cefotaxime (42%), and ampicillin or amikacin (72%). Data regarding susceptibility to ampicillin and gentamicin of 49 neonates were available. A multivariable analysis revealed that patients in the non-susceptible group (*n* = 18) were more likely to experience late-onset EOS (48–72 h; *p* = 0.01) and require endotracheal intubation on day 1 (*p* = 0.04) compared to patients in the susceptible group (*n* = 31). **Conclusions**: In areas with high multidrug resistance, broader-spectrum antibiotic therapy (ampicillin plus amikacin) should be considered for neonates who develop clinical sepsis within 48–72 h of birth and experience respiratory failure at birth.

## 1. Introduction

Neonatal sepsis is associated with mortality and long-term sequelae. Early-onset sepsis (EOS) is defined as sepsis development within 72 h of birth. The American Academy of Pediatrics guidelines recommend empiric therapy comprising a combination of ampicillin and gentamicin for EOS in neonates born before 35 weeks of gestation as well as those born after 35 weeks of gestation [1,2]. The incidence, susceptibility, and outcomes of EOS vary across settings and are dependent on local epidemiology, including different pathogenic isolates, and areas with high multidrug resistance (MDR).

According to prior studies, the overall incidence of neonatal EOS is between 0.5 and 1 per 1000 live births [3,4,5,6,7,8,9,10]. In the United States, the reported case fatality rate (CFR) associated with EOS is between 11.1% and 16.2% [4,6]; however, it is as high as 35.0% for very-low-birth-weight (VLBW) neonates (birth weight [BW] < 1500 g) [11]. In industrialized countries and some developing countries, the most common causative pathogen is group B streptococcus (GBS) [3,6,7,8]. Compared to that in the United States, neonatal sepsis caused by GBS is a rare infection in Southeast Asia [12,13]. Gram-negative organisms such as *Escherichia coli*, *Klebsiella pneumoniae*, and *Acinetobacter baumannii* are the dominant pathogenic isolates in low-income and middle-income countries and countries without an intrapartum antibiotic prophylaxis (IAP) protocol [14,15]. Furthermore, these pathogens are common extended-spectrum beta-lactamase (ESBL)-producing or carbapenem-resistant bacteria [4,16]. Neonatal EOS may lead to increased mortality rates, and a fragile status and non-specific symptoms of neonatal sepsis result in higher rates of antibiotic use [17]. Therefore, antibiotic stewardship programs must be implemented. However, physicians find it difficult to implement these programs in clinical practice. Neonatal mortality may increase if empirical narrow-spectrum antibiotics are prescribed in areas with high MDR; however, broader-spectrum antibiotics may induce colonization or selective pressure of antibiotics.

A recent study performed by the *Eunice Kennedy Shriver* National Institute of Child Health and Human Development Neonatal Research Network (NICHD NRN) revealed that all 81 Gram-positive isolates involved in EOS were susceptible to combination therapy comprising ampicillin and gentamicin [4]. However, some Gram-negative isolates associated with EOS were not susceptible to ampicillin (77%; 72/94), gentamicin (9%; 8/94), or ampicillin plus gentamicin (7%; 7/96) [6]. In areas with high MDR, bacterial isolates may result in a higher rate of non-susceptibility to empirical antimicrobial combination therapy comprising ampicillin and gentamicin for neonatal EOS. Amikacin may be the best therapy for Gram-negative bacteria associated with neonatal sepsis because of its high antimicrobial susceptibility [18]. We compared the susceptibility of second-line therapy (either ampicillin plus cefotaxime or ampicillin plus amikacin) with that of first-line therapy (ampicillin plus gentamicin) for neonatal EOS. However, the risk factors for non-susceptibility to a combination of ampicillin and gentamicin among neonates are limited in areas with high MDR. To determine the clinical characteristics of mothers and neonates who may benefit from altered antimicrobial treatments beyond ampicillin and gentamicin, we compared EOS organisms with and without susceptibility to ampicillin and gentamicin in neonates.

## 2. Results

During the 20-year study period (2004–2023), 131 pathogenic isolates that cause EOS were identified; of these, 71 contaminant organisms were excluded (*Bacillus cereus*, 1; *Bacillus* spp., 12; coagulase-negative *Staphylococci* (CoNS), 7; *Corynebacterium* spp., 1; *Micrococcus luteus*, 1; *Micrococcus* spp., 6; *Rhodococcus equi*, 1; *Staphylococcus cohnii*, 1; *Staphylococcus epidermidis*, 38; *Staphylococcus haemolyticus*, 1; *Staphylococcus warneri*, 1; and *Streptococcus oralis*, 1). One *Bacillus* spp. was included as a pathogen because several positive blood culture results were observed at 0.44 (two blood samples), 0.98, and 2.57 days after birth. Finally, 60 pathogenic isolates (Table 1) and 55 neonatal patients with EOS (including 5 polymicrobial infections) (Table 2) were included in this study. Data regarding susceptibility to ampicillin and gentamicin of 53 pathogenic isolates and 49 neonates were available (Table 3, Table 4 and Table 5).

The five most common causative organisms of EOS were *A. baumannii* (19 [32%]; carbapenem-resistant *A. baumannii* [CRAB]: 12/19 [63%]), *K. pneumoniae* (8 [13%]; ESBL-producer: 6/8 [75%]), *E. coli* (7 [12%]; ESBL-producer: 4/7 [57%]), *Streptococcus agalactiae* (5 [8%]), and *Staphylococcus aureus* (3 [5%]; methicillin-resistant: 1/3 [33%]) (Table 1). Fungal infections were not observed with EOS. During the 20-year period, 62,769 live births occurred. The incidence rates of sepsis caused by *A. baumannii*, *K. pneumoniae*, *E. coli*, *S. agalactiae*, and *S. aureus* were 0.30, 0.13, 0.11, 0.08, and 0.05 per 1000 live births, respectively. The rates of susceptibility of *A. baumannii*, *K. pneumoniae*, *E. coli*, and *S. agalactiae* to the combination of ampicillin and gentamicin were 41%, 50%, 43%, and 80%, respectively. The 7-day mortality rates associated with sepsis caused by *A. baumannii*, *K. pneumoniae*, *E. coli*, *S. agalactiae*, and *S. aureus* were 42%, 13%, 86%, 20%, and 0%, respectively. The most common EOS pathogens in VLBW infants were *A. baumannii* (*n* = 9), *K. pneumoniae* (*n* = 4), *E. coli* (*n* = 4), and *S. agalactiae* (*n* = 2). In the non-susceptible group, all patients with sepsis onset within 24 h (*n* = 3) had positive first blood culture results and all patients with sepsis onset within 24–72 h (*n* = 15) had negative first blood culture results at birth.

The incidence rates of neonates with EOS born at all gestational ages and at ≥34 weeks of gestation were 0.88 (55/62,769) and 0.43 (23/53,906) per 1000 live births, respectively. Of the 55 neonates with sepsis, 2 had both bacteremia and meningitis caused by the same pathogen and antibiogram (*A. baumannii* [*n* = 1] and *E. coli* [*n* = 1]). The incidence rates of EOS among VLBW neonates and extremely low-birth-weight (ELBW) neonates were 1.88% (23/1223) and 3.23% (13/402), respectively. The overall mortality rate, overall CFR, and 7-day CFR among all neonates, VLBW neonates, and ELBW neonates are shown in Table 2.

Data regarding ampicillin and gentamicin susceptibility of 53 of 60 (88.3%) pathogenic isolates and 49 neonates were available (including 4 polymicrobial infections that were positive in the same specimens: *K. pneumoniae* and *Pseudomonas aeruginosa*, *K. pneumoniae* and *E. coli*, *K. pneumoniae* and *A. baumannii*, and *A. baumannii* and *Stenotrophomonas maltophilia*) (Table 3, Table 4 and Table 5). Baseline characteristics of the non-susceptible (37%; 18/49) and susceptible (63%; 31/49) groups are shown in Table 3. Compared with the susceptible group (Table 4), the non-susceptible group was more likely to include smaller neonates (earlier gestational age), those with late-onset EOS (48–72 h), and those who required endotracheal intubation on day 1 of life. All significant variables (*p* < 0.05), including BW, cesarean delivery, antenatal steroid use, pregnancy-induced hypertension, and surfactant administration, were included in the multivariable analysis. After multivariable logistic regression, the non-susceptible group was more likely to develop late-onset EOS (within 48–72 h of birth; adjusted odds ratio, 11.03; 95% confidence interval [CI], 1.79–67.87; *p* = 0.01) and require endotracheal intubation on day 1 of life (adjusted odds ratio, 11.26; 95% CI, 1.14–111.24; *p* = 0.04).

Compared with the susceptible group (Table 5), the non-susceptible group had a higher overall mortality rate (risk difference = 34% [95% CI, 5–56%]; risk ratio = 2.07 [95% CI, 1.03–4.17]) and daily neonatal intensive care unit (NICU) cost (difference in medians = 74.5 United States dollars [USD]).

## 3. Discussion

The local epidemiology of neonatal sepsis is determined based on the incidence, trends during each period, pathogenic isolates, and susceptibility to empirical antimicrobial therapy, as well as the areas with high MDR. Although the incidence of neonatal EOS in this study was similar to that in the United States (1.1 per 1000 live births for term and preterm infants [4] and 1.54% for VLBW infants [19]), pathogens associated with neonatal EOS, susceptibility, and outcomes differed from those of developed countries. These results indicate that physicians should consider switching to a combination of ampicillin and amikacin for neonates with respiratory failure on day 1 of life and clinical sepsis after 48 h, especially in areas with high MDR. Empirical antimicrobial therapy comprising a combination of ampicillin and cefotaxime and meropenem monotherapy may result in poor susceptibility (41.5% and 68.1%, respectively) compared to that associated with ampicillin and amikacin (71.7%). Additionally, carbapenem use may increase colonization pressure and long-term outbreaks of CRAB-related sepsis in areas with high MDR. Finally, some neonates with sepsis initially had negative blood culture results (based on samples from the umbilical catheter after birth) and developed non-susceptible infection within 24–72 h. Therefore, we hypothesized that late-onset clinical manifestation associated with some device-associated pathogens may have originated from horizontal (healthcare-associated) rather than vertical (mother-to-neonate) transmission.

We postulated that pathogenic isolates in areas with high MDR may have been directly transmitted by invasive devices (endotracheal intubation and central line catheterization tubes). Because intubation at birth was identified as a risk factor for non-susceptible infection, and because *A. baumannii*, which was the most common pathogen during this study, colonization was found in water and humidifiers, infection prevention and control (IPC), especially ventilator bundle care, environmental cleaning (sodium hypochlorite cleaning and the use of special ventilator circuits) [17], and antibiotic stewardship programs should be implemented in areas with high MDR.

The overall incidence of EOS was between 0.5 and 1 per 1000 live births according to prior studies [3,4,5,6,7,8,9,10], and it was 0.88 per 1000 live births in this study. The long-term outbreak of *A. baumannii* peaked during 2009 to 2018 (Table 1), and it decreased after IPC interventions were implemented [17]. The incidence of *A. baumannii* sepsis was reduced after IPC interventions in 2014; furthermore, the overall and *A. baumannii* EOS rates decreased during 2019 to 2023. Therefore, EOS prevention (<72 h) can focus on maternal and perinatal transmission (within 24–48 h after birth) and healthcare-associated transmission (48–72 h of birth) by eradicating organism colonization in the NICU in areas with high MDR.

For VLBW infants, the incidence rates of neonatal EOS were 1.34–1.84% according to prior studies [11,20,21]. In this study, their incidence rate of neonatal EOS was 1.88%. The CFRs of EOS among VLBW infants were 35.3% [11] and 56.5% (in this study). The overall mortality rates of VLBW infants were 11.5% in the United States [11] and 15.5% in the present study. In this study, although the mortality rate of VLBW infants was similar to that of those in the United States, the CFR was higher. We postulated that the causative organisms differed at each center, and that the CFR was dependent on the pathogen and susceptibility. Common pathogens observed in VLBW infants were *E. coli* (United States, 41.2%; China, 40.7%; this study, 15.4%), GBS (United States, 11.8%; China, 7.4%; this study 7.7%), and *Haemophilus* species (United States, 2%) [11,21]. However, the common pathogens in this study were *A. baumannii* (34.6%), *K. pneumoniae* (15.4%), and *E. coli* (15.4%).

The risk of EOS among infants born at 34 weeks of gestation or later can be calculated using the Kaiser sepsis calculator. The incidence rates of neonates with EOS born at 34 weeks of gestation or later in Paris, France (2019–2021) [22], in this study, and in the United States (1993–2007) [23] were 0.32 (108/346,162), 0.43, and 0.58 (350/608,014) per 1000 live births, respectively.

Causative pathogens differ among countries. The most common pathogenic isolate associated with EOS was GBS (12.7–45.5% in prior studies and 8.3% in this study) [3,5,6,7,9,24]. Previous studies reported that the incidence of GBS ranged from 0.22 to 0.57 per 1000 live births [6,8,24]; however, in this study it was 0.08 per 1000 live births, which was similar to that observed in Thailand without routine IAP [12,25]. The prevalence of maternal GBS colonization in Thailand was 12–18%; however, in Asian countries and the United States, they were 2–9% and 19–26%, respectively [12]. *E. coli* and *Klebsiella* are common pathogens in developed countries, particularly among preterm infants [5,24,26,27] and low-income and middle-income countries [14,15], respectively. In this study, *A. baumannii* and especially CRAB (63.2%; 12/19) were the most common EOS-related pathogens during 2009 to 2018, when long-term outbreaks of CRAB bacteremia, meningitis [28], ventilator-associated pneumonia, and central line-associated bloodstream infections occurred. In addition to IPC implementation, aggressive extubation, new modes of non-invasive ventilation [29], environmental cleaning, and special respiratory circuit use [17] can reduce CRAB-related sepsis and endotracheal intubation with late-onset sepsis. In addition to perinatal prevention, decreased NICU colonization may decrease the EOS incidence (the incidence during 2019–2023 and that during 2004–2008 were similar, as shown in Table 1).

The rates of susceptibility of pathogenic isolates in Middle Eastern countries [14], this study, and the United States [4], respectively, were as follows: ampicillin, 29%, 30%, and 58%; gentamicin, 57%, 48%, and 91%; third-generation cephalosporins, 57% (cefotaxime or ceftriaxone), 26% (cefotaxime), and 95% (ceftriaxone); amikacin, 56–96%, 68%, and 100%; and carbapenems, 83%, 68%, and 99%. In this study, the rates of susceptibility of the pathogenic isolates to ampicillin or cefotaxime, to gentamicin, and to amikacin or meropenem were low (26–30%), medium (48%), and high (68%), respectively. Additionally, in this study, the rates of susceptibility of EOS pathogenic isolates to ampicillin or gentamicin, to ampicillin or cefotaxime, and to ampicillin or amikacin were 59%, 42%, and 72%, respectively; however, the rates of susceptibility of EOS pathogenic isolates to ampicillin or amikacin were 96% (170/177) in the United States [4] and 98% (99/101) in Norway [8] (Table 1).

The CFR of EOS varies according to countries, trends of the study year, and susceptibility. In the United States, the CFRs during 2002 to 2003, 2005 to 2014, and 2015 to 2017 were 35.0% [11], 11.1% [6], and 16.2% [4], respectively. The CFRs in Asia (2005) [7], Hong Kong (2006–2017) [9], and Norway (1996–2018) [8] were 12.8% (6/47), 9.4% (49/522), and 5.8% (6/101), respectively. In this study, the overall and 7-day CFRs were 41.7% and 34.6%, respectively. We postulated that extreme CFRs were caused by high non-susceptibility rates. The CFRs associated with GBS-related and *E. coli*-related sepsis in this study were higher (20% and 86%, respectively) than those in the United States (7% and 23%, respectively, during 2015–2017) [6].

The multivariable analysis revealed that the non-susceptible group was more likely to experience respiratory failure on day 1 of life and clinical sepsis within 48–72 h. Therefore, broader empirical antimicrobial therapies in areas with high MDR should be considered. According to the univariable analysis results in the United States, the non-susceptible group was more likely than the susceptible group to have an earlier gestational age, lower birth weight, and histologic chorioamnionitis [4]. According to the univariable analysis results of this study, an earlier gestational age and lower BW were significantly associated with non-susceptibility; however, the multivariable analysis did not reveal significance. In the United States and in this study, clinical chorioamnionitis was not significantly associated with non-susceptibility. The non-susceptible group had higher mortality rates at discharge. No differences in the duration of the NICU stay and respiratory support duration were observed because of the short duration of stay of the neonates in the non-susceptible group. However, the daily NICU costs of the non-susceptible group were higher than those of the susceptible group.

This study demonstrated the incidence and susceptibility of EOS as well as risk factors and outcomes of the non-susceptible group in low-income and middle-income countries and areas with high MDR. Some IPC interventions may decrease organisms in NICUs with high CRAB colonization rates in areas with high MDR. A combination of amikacin plus either ampicillin or cefotaxime may be considered as step-up antibiotic treatment for mild-to-moderate critical illness; however, carbapenems are used for severe critical illness or profound septic shock in areas with high MDR after 48 to 72 h of birth. Even in countries where IAP is routinely used, neonates with inadequate IAP exposure (the pathogen yielded in cultures was non-susceptible to the antibiotic administered for IAP) may develop symptoms at ≥48 h of life [30]. Antibiotic stewardship programs and immediate de-escalation based on antibiograms should be implemented to reduce colonization pressure and the need for broader-spectrum antimicrobial therapy.

This study had some limitations. Firstly, although long-term EOS data were collected, several interventions were implemented during each period. Secondly, the histologic chorioamnionitis results were not included because routine pathology tests of the placenta to determine chorioamnionitis were not performed. Thirdly, data regarding maternal, neonatal, device, and environmental surface colonization and microbiomes were lacking. Finally, the sample sizes were small, and wide CIs existed in the multivariable analysis.

## 4. Materials and Methods

### 4.1. Study Population and Data Source

This retrospective study included infants who were born at 22 weeks of gestation or later between 1 January 2004 and 31 December 2023, had a BW > 400 g, and received sepsis treatment in the level IV NICU of Songklanagarind Hospital, which is a teaching and referral hospital affiliated with Prince of Songkla University in Songkhla Province, Thailand. Approximately 2500 to 3500 live births occur at that hospital each year, and approximately 400 to 550 neonates are admitted to the 15-bed NICU annually. This study was approved by the Ethics Committee of the Faculty of Medicine, Prince of Songkla University (REC. 66–497–1–1).

Data regarding maternal and neonatal characteristics and outcomes, culture specimens, organisms, and antimicrobial susceptibility were collected. Antimicrobial susceptibility profiles of Gram-negative organisms considered pathogens were evaluated. Commensal organisms (http://www.cdc.gov/nhsn/xls/master-organism-com-commensals-lists.xlsx, accessed on 14 May 2025) such as *Bacillus* spp., CoNS, *Corynebacterium* spp., *Micrococcus* spp., and *Propionibacterium* spp. were considered pathogens if the neonate had clinical sepsis and received antimicrobial therapy and the results of two or more culture tests were positive for the organism on separate occasions.

### 4.2. Antimicrobial Susceptibility

The antimicrobial susceptibility profiles were classified as susceptible, intermediate, or resistant. Non-susceptibility was defined as intermediate or resistant according to the final antibiogram of the drug or drug group (combination of ampicillin and gentamicin, ampicillin and cefotaxime, or ampicillin and amikacin). If a pathogenic isolate was susceptible to one drug in the combination treatment, then it was considered susceptible. If a pathogenic isolate was intermediate or resistant to two drugs in the combination treatment, then it was identified as non-susceptible.

The antimicrobial susceptibility of each pathogenic isolate in polymicrobial organisms was assessed separately (Table 1). If the same pathogenic organism was isolated from more than one specimen (e.g., two blood cultures or blood and cerebrospinal fluid cultures), then the susceptibility results were evaluated to determine concordance.

An ESBL-producing organism was defined as that with Enterobacterales resistance to third-generation cephalosporins and some second-generation cephalosporins according to the final antibiogram.

### 4.3. Antimicrobial Susceptibility of Individual Sepsis Cases

These cases included pathogenic isolates from single microbial or polymicrobial organisms. If all the polymicrobial pathogenic isolates were susceptible to ampicillin or gentamicin, then they were considered susceptible. If any polymicrobial pathogenic isolates were intermediate or resistant to both drugs, then they were considered non-susceptible.

### 4.4. Empirical Antimicrobial Therapy

The neonatal sepsis protocol for this study involving neonates with both asymptomatic high-risk and symptomatic sepsis included performing a complete blood count, blood culture, and lumbar puncture or urine culture, if indicated, and immediately initiating empirical antibiotics when intravenous access was available. The cutoff for EOS was 72 h after birth, and the antibiotic regimen comprised ampicillin plus gentamicin. Initiating the regimen early and using broader-spectrum empirical antibiotics (e.g., cefotaxime or ceftazidime plus amikacin, cefoperazone/sulbactam, carbapenems, or colistin) may be considered for small preterm infants, moribund neonates, and those with profound hypotension or uncorrectable metabolic acidosis.

Broader-spectrum and narrower-spectrum antimicrobial therapy were adjusted based on whether the clinical condition improved within 48–72 h or the antibiogram. Imipenem, ciprofloxacin, meropenem, cefoperazone/sulbactam, and colistin have been used as empirical antibiotics for severe neonatal sepsis or septic shock during our study since 1992, 2000, 2005, 2007, and 2007, respectively.

### 4.5. Study Definitions and Outcomes

EOS was defined as the isolation of a pathogen from blood and/or cerebrospinal fluid cultures obtained within 72 h of birth. The sepsis date was defined as the first date of clinical sepsis and a positive culture result. ELBW and VLBW were defined as BW < 1000 g and <1500 g, respectively. Premature rupture of membranes was diagnosed more than 18 h before delivery. Maternal antibiotic use was defined as a history of antibiotic use within 7 days before delivery. Pregnancy-induced hypertension was defined as hypertension after 20 weeks of gestation for women with previously normal blood pressure. The Clinical Risk Index for Babies (CRIB) II score predicts neonatal mortality (range, 0−27; higher scores indicate a worse prognosis) [31]. Septic shock was defined as gestational age-dependent hypotension or evidence of poor tissue perfusion that required inotropic agents within 48 h after sepsis onset.

Overall and 7-day mortality were defined as in-hospital mortality and death within 7 days of sepsis onset, respectively. The CFR was calculated by dividing the number of deaths attributable to EOS by the number of individuals diagnosed with EOS. The NICU stay was defined as the duration from admission until discharge from the NICU. The daily NICU cost was calculated as the total NICU cost divided by the total NICU stay (USD 1 = THB 35).

The primary outcome was risk factors for EOS in the non-susceptible group. The secondary outcomes were the incidence of EOS, pathogen type, and outcomes of the non-susceptible and susceptible groups.

### 4.6. Statistical Analysis

R program (version 4.4.1; The R Foundation for Statistical Computing, Vienna, Austria) was used to compare the non-susceptible and susceptible groups. Categorical variables (presented as percentages) were compared using the chi-square test or Fisher’s exact test. Continuous variables were tested for normality (parametric and non-parametric tests) using the Shapiro–Wilk test. Normal and non-normal data distributions (presented as the mean ± standard deviation and median and interquartile range) were compared using Student’s *t*-test and the Wilcoxon rank-sum test, respectively.

Univariable and multivariable analyses were also performed. Independent variables were chosen based on their biological plausibility (*p* < 0.2 in the univariable analysis subjected to generalized linear regression).

## 5. Conclusions

In conclusion, the EOS incidence in areas with high MDR was similar to that in the United States and Asian countries. The most common pathogen related to EOS in this study was *A. baumannii*, especially CRAB. Susceptibility to the recommended combination of ampicillin and gentamicin was low (57%), and the probability of empirical antibiotic coverage was arbitrary. Empirical antimicrobial therapy comprising a combination of ampicillin and amikacin should be considered for neonates with clinical sepsis development within 48–72 h of birth and a history of respiratory failure at birth in areas with high MDR. Clinical practitioners should focus on an appropriate evaluation of local antibiograms and local bacterial resistance patterns when considering a shift in empirical antibiotic usage; additionally, they should highlight the use of risk stratifications to enhance antibiotic stewardship. Therefore, studies of local epidemiology in low-income and middle-income countries, local protocols, active surveillance, neonatal IPC interventions, and new therapies beyond antibiotics (e.g., obligate living bacterial predators of other bacteria, including ESBL and carbapenemase producers and colistin-resistant pathogenic isolates) are required.

## Figures and Tables

**Table 1:** Isolates and antimicrobial susceptibility rates of organisms that cause early-onset sepsis.

Organism	Isolates (*n*)					Susceptible Organisms (*n*)												
	2004–2023	2004–2008	2009–2013	2014–2018	2019–2023	AM or GM	AM or CTX	AM or AK	AK	AM	CTX	CL	GM	IMP	MEM	OX	PG	VA
**Gram-positive**	18	5	4	6	3	12/14	12/14	12/14	2/2	12/14	0/2	0/0	4/5	7/8	2/2	2/3	11/13	10/11
*Streptococcus agalactiae*	5	1	1	2	1	4/5	4/5	4/5		4/5				1/1			4/5	2/2
*Staphylococcus aureus*	3	1	1	1	0											2/3		3/3
*Streptococcus* beta (not groups A, B, and D)	2	1	1	0	0	2/2	2/2	2/2		2/2			1/1	1/1			2/2	1/1
*Streptococcus* gamma (not group D)	2	1	1	0	0	2/2	2/2	2/2		2/2				2/2			2/2	2/2
*Enterococcus* spp.	2	0	0	1	1	1/2	1/2	1/2		1/2			1/2	1/2			1/2	1/2
*Listeria monocytogenes*	2	0	0	2	0	2/2	2/2	2/2	2/2	2/2	0/2		2/2	2/2	2/2		1/1	1/1
*Bacillus* spp.	1	1	0	0	0													
*Bifidobacterium longum*	1	0	0	0	1	1/1	1/1	1/1		1/1							1/1	
**Gram-negative**	42	3	15	16	8	19/39	10/39	26/39	24/36	4/39	10/36	32/32	16/37	25/39	26/39			
*Acinetobacter baumannii*	19	0	10	7	2	7/17	2/17	7/17	7/17	1/17	2/16	16/16	7/17	7/19	7/19			
*Klebsiella pneumoniae*	8	0	2	2	4	4/8	2/8	7/8	7/8	0/8	2/8	7/7	3/8	8/8	8/8			
*Escherichia coli*	7	1	1	4	1	3/7	2/7	6/7	6/6	0/7	2/6	5/5	3/7	7/7	7/7			
*Enterobacter cloacae*	2	0	0	1	1	1/2	1/2	2/2	2/2	0/2	1/2	2/2	1/2	2/2	2/2			
*Haemophilus influenzae*	2	2	0	0	0	2/2	2/2	2/2		2/2	2/2							
*Acinetobacter junii*	1	0	0	1	0	1/1	1/1	1/1	1/1	1/1	1/1	1/1	1/1	1/1	1/1			
*Neisseria* spp.	1	0	1	0	0													
*Pseudomonas aeruginosa*	1	0	0	1	0	1/1	0/1	1/1	1/1	0/1		1/1	1/1	0/1	1/1			
*Stenotrophomonas maltophilia*	1	0	1	0	0	0/1	0/1	0/1	0/1	0/1	0/1		0/1	0/1	0/1			
Total (*n*)	60	8	19	22	11	31/53	22/53	38/53	26/38	16/53	10/38	32/32	20/42	32/47	28/41	2/3	11/13	10/11
Total (%)						58.5	41.5	71.7	68.4	30.2	26.3	100	47.6	68.1	68.3	66.7	84.6	90.9

AK, amikacin; AM, ampicillin; CTX, cefotaxime; CL, colistin; GM, gentamicin; IMP imipenem; MEM, meropenem; OX, oxacillin; PG, penicillin; VA, vancomycin.

**Table 2:** Overall mortality and case fatality rates.

Population	Overall Mortality Rate, % (*n*)	Overall Case Fatality Rate, % (*n*)	7-Day Case Fatality Rate, % (*n*)
All neonates	0.68 (429/62,769)	41.8 (23/55)	34.6 (19/55)
Infants with very low birth weight	15.5 (189/1223)	56.5 (13/23)	43.5 (10/23)
Infants with extremely low birth weight	30.1 (121/402)	76.9 (10/13)	61.5 (8/13)

**Table 3:** Characteristics of neonates with early-onset sepsis in the susceptible and non-susceptible groups.

Characteristics	Non-Susceptible Group (*n* = 18)	Susceptible Group (*n* = 31)	*p*-Value
Gestational age, weeks *	29.9 ± 4.2	33.3 ± 5.0	0.02
Birth weight, grams †	1330 (915–1715)	1515 (1133–2858)	0.14
Sepsis onset, *n* (%)			0.02
Within 24 h	3 (16.7)	17 (54.8)	0.02
24–48 h	7 (38.9)	9 (29)	0.69
48–72 h	8 (44.4)	5 (16.1)	0.045
Study year of neonatal sepsis, *n* (%)			0.53
2004–2013	10 (55.6)	13 (41.9)	
2014–2023	8 (44.4)	18 (58.1)	
Male, *n* (%)	8 (44.4)	20 (64.5)	0.29
Small for gestational age, *n* (%)	0 (0)	8 (25.8)	0.02
Extremely low birth weight, *n* (%)	7 (38.9)	6 (19.4)	0.18
Cesarean delivery, *n* (%)	14 (77.8)	15 (48.4)	0.09
Premature rupture of membranes, *n* (%)	3 (16.7)	5 (16.1)	1.00
Clinical chorioamnionitis, *n* (%)	1 (5.6)	3 (9.7)	1.00
Cervical cerclage or obstetric procedure, *n* (%)	1 (5.6)	0 (0)	0.37
Maternal antibiotics, *n* (%)	4 (22.2)	5 (16.1)	0.71
Antenatal steroids, *n* (%)	11 (61.1)	10 (32.3)	0.10
Pregnancy-induced hypertension, *n* (%)	5 (27.8)	3 (9.7)	0.12
Delivery resuscitation, *n* (%)			0.83
Routine care	5 (27.8)	13 (41.9)	
Positive pressure ventilation	10 (55.6)	13 (41.9)	
Chest compression	1 (5.6)	2 (6.5)	
Epinephrine administration	2 (11.1)	3 (9.7)	
5 min Apgar score †	8 (6–9)	9 (8–9)	0.20
Clinical Risk Index for Babies II score *	7.7 ± 4.2	7.4 ± 3.9	0.80
Endotracheal intubation on day 1 of life, *n* (%)	17 (94.4)	20 (64.5)	0.04
Surfactant administration, *n* (%)	9 (50)	8 (25.8)	0.16
Meconium aspiration syndrome, *n* (%)	1 (5.6)	4 (12.9)	0.64
Septic shock ‡, *n* (%)	11 (61.1)	10 (32.3)	0.10

* Data are presented as the mean ± standard deviation. † Data are shown as the median (interquartile range). ‡ On the date of sepsis onset.

**Table 4:** Univariable and multivariable logistic regression analyses of risk factors for early-onset sepsis in neonates in the susceptible and non-susceptible groups.

Risk Factors	Univariable Analysis		Multivariable Analysis	
	Crude OR (95% CI)	*p*-Value	Adjusted OR (95% CI)	*p*-Value
Gestational age	0.86 (0.73–0.97)	0.02		
Birth weight	0.9994 (0.9987–1)	0.07		
Sepsis onset				
Within 24 h	Reference = 1		Reference = 1	
24–48 h	4.41 (0.97–24.60)	0.07	4.18 (0.81–21.60)	0.09
48–72 h	9.07 (1.88–55.65)	0.009	11.03 (1.79–67.87)	0.01
Cesarean delivery	0.27 (0.06–0.94)	0.04		
Antenatal steroid	3.3 (1.01–11.58)	0.05		
Pregnancy-induced hypertension	3.59 (0.77–19.75)	0.11		
Endotracheal intubation on day 1 of life	9.35 (1.57–179.84)	0.04	11.26 (1.14–111.24)	0.04
Surfactant administration	2.87 (0.85–10.13)	0.09		

CI, confidence interval; OR, odds ratio.

**Table 5:** Outcomes of neonates with early-onset sepsis in the susceptible and non-susceptible groups.

Characteristics	Non-Susceptible Group (*n* = 18)	Susceptible Group (*n* = 31)	*p*-Value
Overall mortality rate, *n* (%)	12 (67)	10 (32)	0.04
7-day mortality rate, *n* (%)	9 (50)	9 (29)	0.25
Duration of NICU stay, days *	4 (3–17.8)	5 (3–19)	0.65
Daily NICU cost, USD *	USD 282 (USD 208–USD 528)	USD 208 (USD 99–USD 274)	0.04

* Data are presented as the median (interquartile range). NICU, neonatal intensive care unit; USD, United States dollars.

## Data Availability

The raw data that support the conclusions of this study are available from the authors on request.

## Abbreviations

CFR	Case fatality rate
CRAB	Carbapenem-resistant *A. baumannii*
ELBW	Extremely low birth weight
EOS	Early-onset sepsis
ESBL	Extended-spectrum beta-lactamase
IAP	Intrapartum antibiotic prophylaxis
GBS	Group B streptococcus
MDR	Multidrug resistance
NICU	Neonatal intensive care unit
VLBW	Very low birth weight
